# Inspiratory Capacity during Exercise: Measurement, Analysis, and Interpretation

**DOI:** 10.1155/2013/956081

**Published:** 2013-02-07

**Authors:** Jordan A. Guenette, Roberto C. Chin, Julia M. Cory, Katherine A. Webb, Denis E. O'Donnell

**Affiliations:** ^1^Department of Physical Therapy, University of British Columbia, Vancouver, BC, Canada V6T 1Z3; ^2^UBC James Hogg Research Centre, Institute for Heart + Lung Health, St. Paul's Hospital, Vancouver, BC, Canada V6Z 1Y6; ^3^Respiratory Investigation Unit, Department of Medicine, Queen's University and Kingston General Hospital, Kingston, ON, Canada K7L 2V7

## Abstract

Cardiopulmonary exercise testing (CPET) is an established method for evaluating dyspnea and ventilatory abnormalities. Ventilatory reserve is typically assessed as the ratio of peak exercise ventilation to maximal voluntary ventilation. Unfortunately, this crude assessment provides limited data on the factors that limit the normal ventilatory response to exercise. Additional measurements can provide a more comprehensive evaluation of respiratory mechanical constraints during CPET (e.g., expiratory flow limitation and operating lung volumes). These measurements are directly dependent on an accurate assessment of inspiratory capacity (IC) throughout rest and exercise. Despite the valuable insight that the IC provides, there are no established recommendations on how to perform the maneuver during exercise and how to analyze and interpret the data. Accordingly, the purpose of this manuscript is to comprehensively examine a number of methodological issues related to the measurement, analysis, and interpretation of the IC. We will also briefly discuss IC responses to exercise in health and disease and will consider how various therapeutic interventions influence the IC, particularly in patients with chronic obstructive pulmonary disease. Our main conclusion is that IC measurements are both reproducible and responsive to therapy and provide important information on the mechanisms of dyspnea and exercise limitation during CPET.

## 1. Introduction

Cardiopulmonary exercise testing (CPET) is increasingly recognized as an important clinical diagnostic tool for assessing exercise intolerance and exertional symptoms, and for objectively determining functional capacity and impairment [1]. CPET is particularly well suited for understanding factors that may limit or oppose (i.e., constrain) ventilation in the face of increasing ventilatory requirements during exercise both in research and clinical settings. Traditionally, ventilatory reserve has been evaluated by examining the relationship between peak exercise ventilation (*V*_*E*_) and the measured (or estimated) maximal voluntary ventilation (MVV). Thus, an increased ratio (e.g., *V*_*E*_/MVV >  ~85%) occurring at a relatively low work rate, in the setting of an adequate cardiovascular reserve, strongly suggests that ventilatory factors are contributing to exercise limitation [1]. However, MVV may not accurately reflect sustainable peak *V*_*E*_ in some individuals since respiratory muscle recruitment patterns, operating lung volumes, breathing pattern, and respiratory sensation are distinctly different during brief bursts of voluntary hyperpnea compared with the hyperpnea of exercise [2]. Moreover, the ventilatory reserve provides little information on the factors that limit or constrain further increases in *V*_*E*_ [3] or, indeed, the concomitant sensory implications. It is increasingly clear that perceived intolerable respiratory discomfort may limit exercise even before physiological maxima are reached and needs to be considered in CPET interpretation. 

**Figure 1:**
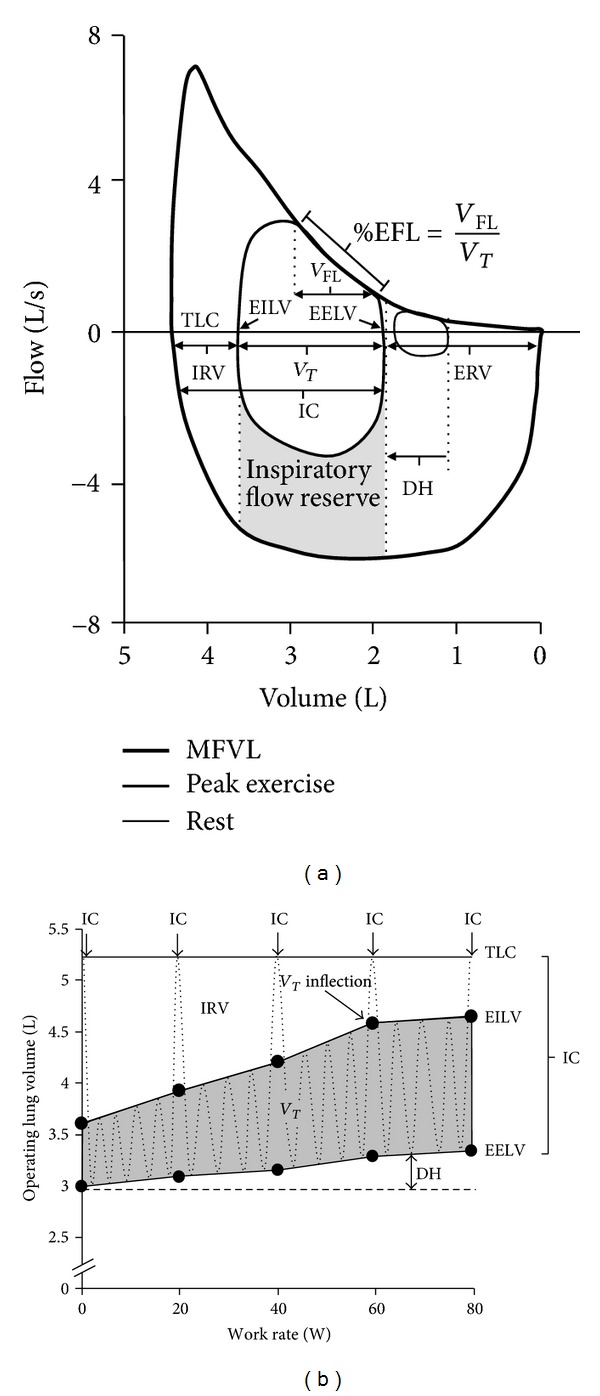
(a) Example of a resting and peak exercise tidal breath superimposed within a maximum flow-volume loop (thick black line). Modified from [3]. The position of the tidal breaths along the *x*-axis is based on the measurement of end-expiratory lung volume (determined from inspiratory capacity maneuvers). (b) Operating lung volume plot versus cycle work rate. Inspiratory capacity maneuvers are performed at rest (0 W) and every 20 W throughout exercise. TLC, total lung capacity; IRV, inspiratory reserve volume; EILV, end-inspiratory lung volume; EELV, end-expiratory lung volume; *V*_*T*_, tidal volume; IC, inspiratory capacity; *V*_FL_, volume of the tidal breath that is flow limited on expiration; %EFL, percentage of expiratory flow limitation; ERV, expiratory reserve volume; MFVL, maximum flow-volume loop; DH, dynamic hyperinflation.

More detailed assessments during CPET can provide additional valuable information regarding the presence of respiratory mechanical constraints to ventilation. For example, Johnson et al. [3] have advocated the flow-volume loop analysis technique for estimation of both inspiratory and expiratory flow reserves during exercise in health and in cardiopulmonary disease. This approach has proven clinical utility: it permits the estimation of expiratory flow limitation, the extent of dynamic hyperinflation, and tidal volume (*V*_*T*_) constraints [3] (Figure 1(a)). However, it is important to consider the potential confounding effects of thoracic gas compression and bronchodilation when using this technique [4]. Another refinement in the assessment of mechanical volume constraints is the portrayal of changes in operating lung volumes (*V*_*T*_, end-expiratory lung volume (EELV), end-inspiratory lung volume (EILV), and inspiratory reserve volume (IRV)) as a function of time, *V*_*E*_, work rate or oxygen uptake (*V*_O_2__) during exercise (Figure 1(b)). This approach has the advantage of graphically displaying the time course of change in all of the relevant operating lung volumes throughout exercise relative to total lung capacity (TLC). This analysis of operating lung volumes, in conjunction with breathing pattern and dyspnea intensity ratings, allows a comprehensive evaluation of ventilatory abnormalities during exercise and their contribution to exercise limitation in the individual patient. Both of these approaches are critically dependent on an accurate measurement of inspiratory capacity (IC) to track changes in EELV. EELV can also be measured using gas dilution techniques [5], respiratory inductance plethysmography [6], or optoelectronic plethysmography [7]. However, these technically demanding methods are expensive, they require specialized training, and they are rarely used in clinical settings. The simplest and most widely accepted method for measuring EELV during exercise is to have individuals perform serial IC maneuvers at rest and throughout exercise [4, 8–12]. A number of software options are now available on various commercial metabolic measurement systems to facilitate such measurements during CPET.

The IC, the maximal volume of air that can be inhaled after a quiet breath out, is a relatively simple measurement and it does not require any specialized equipment since all metabolic systems are able to measure lung volume. Despite the simplicity of this measurement, the IC provides valuable information on the ventilatory response to exercise; it is often used as a primary or secondary endpoint in clinical trials [13–15]; and it correlates well with several important outcome parameters such as peak *V*_O_2__ [16, 17] and carbon dioxide retention during exercise [18]. When expressed relative to TLC, the resting IC is an independent risk factor for mortality [19] and acute exacerbation [20] in patients with chronic obstructive pulmonary disease (COPD). Progressive reductions in the resting IC with increasing COPD severity have also been shown to be associated with important mechanical constraints on *V*_*T*_ expansion and the development of dyspnea during exercise [12]. In addition, dynamic lung hyperinflation, defined as the temporary and variable increase of EELV above the resting value, can contribute importantly to dyspnea and exercise intolerance in patients with chronic lung disease [17]. Other important consequences associated with dynamic hyperinflation include (1) increased elastic and threshold loading on the inspiratory muscles resulting in an increased work and O_2_ cost of breathing; (2) *V*_*T*_ constraints resulting in early mechanical ventilatory limitation; (3) functional inspiratory muscle weakness and possible fatigue; (4) CO_2_ retention and arterial O_2_ desaturation; (5) adverse effects on cardiac function (see Table 1 and [21]). Dynamic hyperinflation can be tracked as a progressive reduction in IC during exercise. Despite the well-known association between static and dynamic IC and its role in the genesis of dyspnea and exercise intolerance, there are no specific guidelines or recommendations on how to adequately perform, analyze, and interpret the IC, particularly during exercise. Given the valuable clinical and research insight that this measurement can provide, a standardized approach to this method is warranted. Accordingly, the purpose of this paper is to critically evaluate the method of measuring IC during exercise. Specifically, we will address issues related to methodological assumptions and reproducibility of the IC, how to perform the maneuver, and how to analyze and interpret IC data. This paper will also briefly address typical IC responses to exercise in health and disease. We will evaluate the utility of assessments of dynamic operating lung volumes and breathing pattern to assess mechanical constraints to ventilation and discuss the effects of various therapeutic interventions on the IC at rest and during exercise in patients with COPD.

## 2. Assumptions and Reproducibility

Accurate assessment of EELV (calculated as TLC minus IC) is directly dependent on the stability of TLC throughout exercise and the ability of the individual to maximally inflate their lungs during the IC maneuver. Thus, if TLC is constant, then any change in IC will reflect the inverse change in EELV. Constancy of TLC has been demonstrated during exercise in healthy individuals [22] and in patients with COPD [23]. It also appears that individuals with COPD are able to maximally activate their diaphragm during inspiratory efforts to TLC [24, 25], even when dyspneic at peak exercise [24]. Yan et al. [26] determined the reliability of IC measurements in individuals with COPD during incremental cycle exercise by comparing esophageal pressure at peak inspired plateau volume during serial IC efforts. These authors demonstrated consistent peak esophageal pressures throughout exercise despite changes in IC. They concluded that TLC did not change and that the IC was reliable for assessing changes in EELV during exercise. This conclusion is supported by other studies which have shown high reproducibility of the IC [10, 27] and its responsiveness to change during exercise following different forms of therapy [28–32]. O'Donnell et al. [33] recently extended these observations by examining reproducibility of the IC at rest and during cycle exercise in large multicentre clinical trials. These authors demonstrated high reproducibility of the IC at rest, isotime, and at peak exercise (intraclass correlation *R* ≥ 0.87). Reproducibility data of IC measurements during treadmill exercise or walk tests have not been published to date. 

To our knowledge, no information is available about the reliability of IC measurements to track operating lung volumes in other clinical populations. For example, reductions in IC during exercise have been reported in obesity [34], congestive heart failure [35, 36], pulmonary arterial hypertension [37], and cystic fibrosis [38]. Since inspiratory muscle weakness may be present to a variable degree in some, if not all, of these conditions, the assumption that IC reduction during exercise represents an increase in EELV must be made with caution. Accurate interpretation of IC behaviour in these circumstances requires the concomitant assessment of respiratory muscle function and peak inspiratory pressures during the IC maneuver. 

## 3. Measurement of IC

### 3.1. Technical Considerations

**Figure 2:**
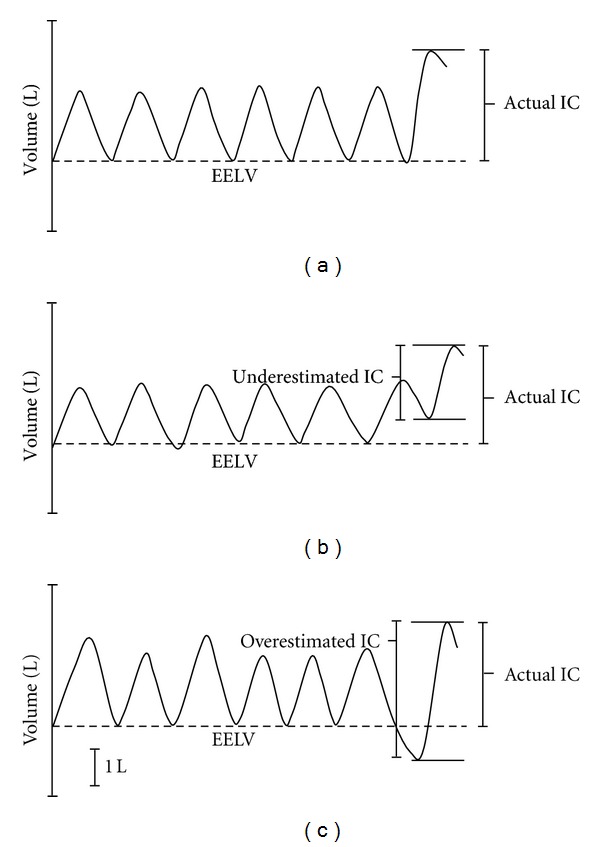
Three examples of inspiratory capacity (IC) maneuvers performed during exercise. (a) Correctly performed maneuver where the individual initiates the IC maneuver at the appropriate end-expiratory lung volume (EELV), denoted by the dashed line. (b) Individual initiating the IC maneuver prior to reaching the appropriate EELV. The absolute volume of the IC breath will be underestimated if it is not anchored to the appropriate EELV. (c) Individual initiating the IC maneuver after surpassing the appropriate EELV. The absolute volume of the IC breath will be overestimated if it is not anchored to the appropriate EELV.

The ability to accurately evaluate IC during exercise requires the measurement of bidirectional flow using flow sensing devices, which is then integrated to calculate volume. Metabolic carts that only measure inspiratory flow are inappropriate for measuring IC. This is because many individuals will alter their breathing pattern prior to performing an IC maneuver. Specifically, they either decrease or increase their expired volume immediately prior to the IC resulting in an underestimation or overestimation of IC, respectively (Figure 2). Depending on the measurement tool and method of delivery of instructions, there can also be anticipatory changes in breathing pattern that can increase the variability of premaneuver EELV. It is therefore essential that inspiratory and expiratory volumes be continuously monitored so that alterations in EELV can be identified and accounted for (see Section 4).

An important technical consideration when measuring bidirectional flow/volume is that signal “drift” occurs with all flow sensing devices. Drift may occur as a result of electrical changes over time, nonlinearities in the flow sensing device, and physiological changes such as temperature, gas density, and humidity [39]. Drift must therefore be accounted for prior to analysis of the IC maneuver [3, 27]. Most commercially available breath-by-breath metabolic systems that offer exercise flow-volume analysis software account for thermodynamic drift by correcting both the inspiratory and expiratory flow/volume signals to BTPS conditions. The tester should be able to view the volume-time plot in real-time during the maneuvers to monitor changes in breathing pattern and drift. Real-time assessments of changes in EELV using tidal flow-volume plots are also popular but, in our experience, may be more difficult than volume-time plot analysis. 

There is limited information regarding standards for intermaneuver reproducibility of resting IC measurements. The American Thoracic Society and European Respiratory Society Task Force [40] simply states that there should be at least three acceptable maneuvers and that the mean coefficient of variation for IC is 5 ± 3% in patients with chronic airflow obstruction, based on the work of Pellegrino et al. [41]. Although they suggest that the two largest acceptable values of other spirometric values (forced vital capacity (FVC) and forced expiratory volume in 1 second) should be within 0.15 L of each other, or within 0.10 L if FVC is ≤1.0 L, they do not provide specific criteria for IC. The American Association for Respiratory Care suggests that IC measurements should agree within 5% or 60 mL of the mean (whichever is larger) [42]. However, the 5% or 60 mL cutoff may be too stringent for resting IC measurements. If patients are unable to achieve reasonable reproducibility at rest, then it is unlikely that they will be able to accurately perform IC measurements during exercise.

### 3.2. Exercise Protocols

A wide range of protocols on both treadmills and cycle ergometers have been used for the evaluation of IC during exercise, including constant work rate tests [14, 43, 44] and incremental tests [9, 17, 28, 45]. There does not appear to be a major difference in IC values when comparing treadmill versus cycle exercise [46, 47], at least in patients with COPD. The duration of each exercise stage can vary for incremental exercise tests depending on the population and the purpose of the study (e.g., 1–3 minute stages). The main consideration when selecting exercise protocols, particularly for incremental tests, is to use stepwise increases in work rates. Ramp tests, where the work rate incrementally increases every 1-2 seconds, are probably inappropriate for measuring IC due to the inability to establish stable ventilations. IC maneuvers are typically performed during the final 30 seconds of each exercise stage when *V*_*E*_ is assumed to be reasonably stable. 

### 3.3. Performing the IC Maneuver

The IC maneuver involves a maximal inspiration from a stable EELV to TLC. Despite the relative simplicity of this technique, several steps must be taken to ensure optimal performance by the individual. Like any volitional test, we have to assume that individuals are able to give a true maximal effort for the IC value to be accurate. Careful and consistent instructions are critically important and testers must be appropriately trained in explaining the maneuver to the individual. Individuals should be given sufficient time to practice the maneuvers at rest and during exercise for familiarization purposes. It is important to first explain the maneuver in general terms to the individual and to heavily emphasize the importance of fully inflating their lungs. It is then recommended that the tester demonstrate the test with an emphasis on the volitional nature of the maneuver. The following is an example of general instructions: “*During the resting period and once during every stage of exercise, we are going to ask you to take a deep breath in until you are completely full. To do this, you will finish your normal breath out and then proceed to fill up your lungs quickly and without hesitation until you are as full as possible. When you are certain you can't get any more air in then you can go back to normal breathing.*”

When the individual is breathing on the mouthpiece at rest and their breathing pattern is stable, then the following (or similar) instructions should be given to prompt the initiation of the IC maneuver: *“at the end of a normal breath out, take a deep breath all the way in until you are completely full.”* During the IC maneuver, the tester should give verbal encouragement (e.g., *“in in in…”*). The tester should also encourage the individual to avoid holding their breath during the maneuver. Ideally, the tester should be able to view the volume-time trace and/or the flow-volume loop tracing during and after the maneuver. If the individual does not initiate the IC at a stable EELV then it is recommended that the tester reexplain what is meant by *“at the end of a normal breath out.”* Doing this during the familiarization period is most appropriate. There is a natural tendency for some individuals to “cheat” immediately before performing the IC maneuver by taking a smaller or larger tidal breath out than the previous stable breaths as shown in Figure 2. Giving the individual visual feedback on their test at rest or even drawing out an example during the familiarization period may help some individuals better understand what is meant by *“at the end of a normal breath out*.” 

It is also important to note that some individuals take several more breaths before performing the maneuver once the prompt is given by the tester. Some of these individuals significantly change their breathing pattern (rate and depth) as an anticipatory response to performing the IC. In some cases, individuals will even alter their cadence if they are on the cycle ergometer. In these instances, it should be encouraged that the individual not *“over-think”* the test and try to perform the IC as soon as they are given the prompt to do so. The alternative is to tell the individual when to perform the IC (i.e., *“at the end of this (the next) breath out, take a deep breath all the way in until you are completely full”*). In rare instances where individuals struggle with both of these approaches, the tester may consider telling them to maximally inspire without any warning. This approach requires careful monitoring of flow and volume tracings and/or watching the individual's breathing rhythm. As soon as the tester sees that the individual is about to take a breath in, they can quickly tell them to maximally inflate their lungs: *“all the way in on this breath – in in in…”* However, this approach is extremely difficult if breathing frequency is very high. The ideal situation is to have the instructions and method standardized for all individuals. However, alternative approaches must be used if the individual has difficulty following instructions or has major alterations in breathing pattern when given the prompt to perform the IC.

It should be noted, however, that if the breathing pattern alterations immediately prior to the IC maneuver are relatively minor, then the data can still be used as long as the baseline EELV is adjusted according to the stable breaths prior to the IC. It is therefore critical that there is stable breathing for at least 4 breaths prior to the IC. This is not a problem for many individuals (particularly during exercise), but some individuals find the mouthpiece uncomfortable and they will often cough, swallow, or clear their throat. For these individuals, it may be appropriate to remind them to avoid coughing or swallowing when stable breathing patterns are most important for data collection. 

Obtaining a reliable IC at peak exercise can also be a challenge. The most accurate peak exercise IC is that obtained immediately prior to exercise cessation. Performing the peak exercise IC several breaths into recovery is usually not appropriate given that the breathing pattern typically changes immediately upon reducing the work rate and since IC may quickly return to resting levels after exercise cessation. This can be challenging if the individual terminates exercise suddenly. Accordingly, we recommend that testers give the following instructions during the preexercise resting period: *“The goal is for you to exercise as long as you can until you feel like you can't go any longer. When you feel like you have about 10 seconds left, give us a warning wave with your hand so that we can get you to perform the last breathing maneuver.”* We recommend giving them a reminder when the exercise test is becoming more difficult using the following (or similar instructions): *“as a quick reminder, when you feel like you can't go any longer, just give us a 10 second warning wave.”* Then immediately say: *“you're still looking really strong though so keep going for as long as you can.”* This motivational statement is important because some individuals will use the 10 second warning reminder as an invitation to stop exercising. As soon as the individual gives the warning wave, provide verbal encouragement: *“you're almost there…only a few seconds left…keep going.”* Once enough tidal breaths are recorded, have the subject perform the IC and then immediately reduce the exercise load. 

## 4. Analysis of IC

The first step in analyzing IC data is to ensure that drift in the volume-time trace has been adequately corrected [3, 27]. The tester then needs to decide if the IC maneuver should be accepted or rejected. The following general guidelines should be used to establish if the IC should be rejected.

- *Number of Premaneuver Tidal Breaths Available for the Assessment of EELV*. It is recommended to have a minimum of 4 stable breaths prior to the IC maneuver in order to accurately establish the baseline EELV (Figure 2). The best approach is to continuously monitor volume so that all breaths are captured. However, some commercially available systems that offer IC modules only permit data collection for a defined time period (e.g., 30 seconds). This 30-second time limit may be inappropriate, particularly if breathing frequency is very low.
- *Variability of EELV Prior to the IC Maneuver*. Too much variability in EELV could be due to anticipatory changes in breathing pattern and/or excessive drift due to moisture accumulation in the flow sensor and/or air leaks at the mouth/nose. Anticipatory changes in breathing pattern can be identified during the test by the tester. With adequate instruction and practice by the individual, this problem can generally be avoided. Excessive signal drift due to imperfect correction of inspiratory and expiratory flow signals to BTPS conditions, or due to moisture accumulation, may be difficult to correct and may result in spurious IC values. Leaks at the mouth can also be avoided by reminding the individual to ensure that they have a good seal around the mouthpiece throughout the test.
- *Adequacy of Inspiratory Effort.* Accurate assessment of inspiratory effort can be accomplished by simultaneously measuring peak inspiratory esophageal pressure during the IC maneuver [26, 48]. If peak inspiratory pressures during exercise are similar to the pressures obtained repeatedly at rest during the IC maneuver, then it is safe to assume adequate effort. However, esophageal pressure measurements are invasive and not necessary for most clinical- and research-based exercise tests. Quantification of effort without esophageal pressure can be difficult. However, providing verbal encouragement during the IC maneuver and emphasizing the volitional nature of the test during the instruction period can be helpful to ensure adequate effort. Finally, simple observation of the individual during the IC maneuver will often allow the tester to determine if the effort was appropriate.

If a test is deemed adequate for analysis (i.e., stable premaneuver breathing pattern, stable premaneuver EELV, and good inspiratory effort to TLC), then the tester can establish the baseline EELV. The volume during the IC breath minus the baseline EELV value represents the IC volume (Figure 2). Establishing the baseline EELV can be automated or manually determined. Manual adjustment is offered on some commercially available systems (i.e., by dragging a horizontal line on the volume-time plot or a vertical line on the flow-volume plot to the appropriate EELV). This approach is subjective and could be affected by tester bias. Thus, for research-related testing, it is appropriate for the tester to be blinded to the experimental conditions in order to avoid introducing possible bias into the analysis. 

### 4.1. Measurements Derived from IC Maneuvers

**Figure 3:**
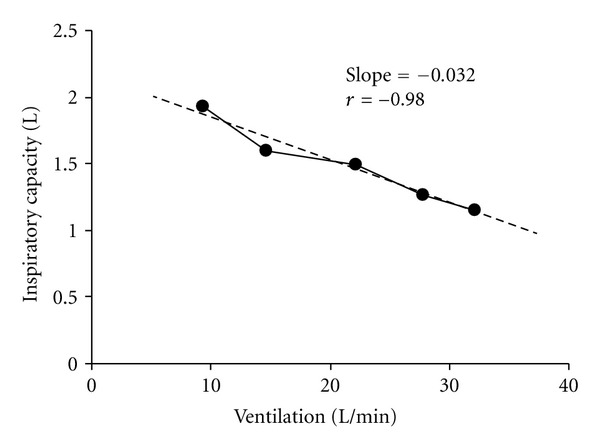
Dynamic hyperinflation can be evaluated as the linear slope relating inspiratory capacity and minute ventilation [49]. Dynamic hyperinflation is typically assessed at a single time point during an exercise test. The slope method accounts for all inspiratory capacity measurements during an exercise test and takes into account possible changes in ventilation that can occur with various interventions (e.g., hyperoxia, heliox, bronchodilators, exercise training, etc.).

Table 2 shows the range of variables that can be derived from IC measurements collected at rest and during exercise, and the various ways in which these variables can be expressed. For example, dynamic hyperinflation can be evaluated as the difference between the IC at rest and during exercise (ΔIC). The same value will be obtained if you take the difference between EELV at rest and during exercise. These approaches provide information regarding the magnitude of dynamic hyperinflation at a single time point during exercise. An alternative to evaluating dynamic hyperinflation at one time point is to examine the slope relating the full range of IC values to *V*_*E*_ at rest and throughout exercise [10, 49] (Figure 3). This approach takes into account all data points and any changes in *V*_*E*_ that might occur with different interventions (e.g., hyperoxia and exercise training). However, the slope approach to analysis may not be appropriate in all cases since changes in IC may not always change linearly with *V*_*E*_. 

**Figure 4:**
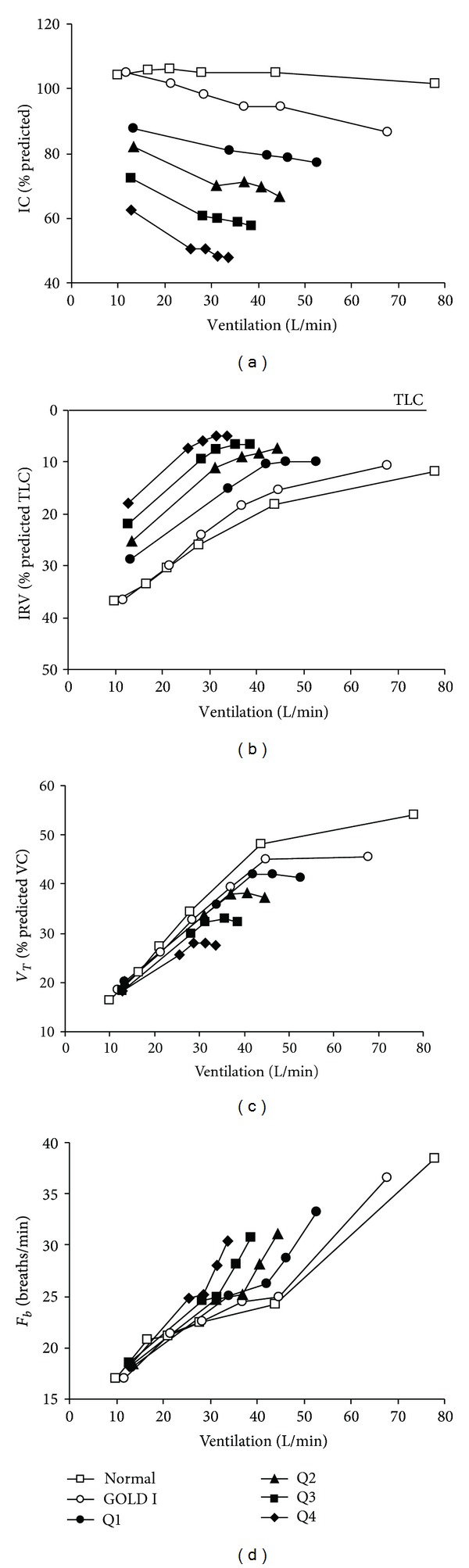
Inspiratory capacity (IC), inspiratory reserve volume (IRV), tidal volume (*V*_*T*_), and breathing frequency (*F*_*b*_) responses versus minute ventilation during constant work rate exercise across the continuum of health and COPD severity. The IC at rest and throughout exercise progressively decreases with advancing disease. Note the clear inflection (plateau) in the *V*_*T*_-ventilation relationship, which coincides with a simultaneous inflection in the IRV-ventilation relationship. After this point, further increases in ventilation are accomplished by accelerating *F*_*b*_. Data from Normal subjects and GOLD stage I (i.e., mild COPD) are from Ofir et al. [61]. Quartiles (Q) of COPD severity are based on forced expiratory volume in 1 second (FEV_1_) expressed as percent predicted (ranges: Q1 = 54.5–85.1; Q2 = 43.8–54.1; Q3 = 34.9–43.6; Q4 = 16.5–34.9) from O'Donnell et al. [12]. VC, vital capacity; TLC, total lung capacity; GOLD, Global Initiative for Obstructive Lung Disease.

Operating lung volumes can provide valuable insight into the respiratory response to exercise. Similar to the flow-volume loop approach (Figure 1(a)), operating volume plots (Figure 1(b)) allow the researcher or clinician to examine the EELV and EILV, the magnitude of dynamic hyperinflation, the presence of *V*_*T*_ constraints, and the inspiratory and expiratory reserve volumes. Accurate measurement of operating volumes in absolute terms (litres) is dependent on the measurement of TLC. However, some laboratories are only capable of measuring FVC (or vital capacity (VC)). In these cases, a surrogate for EELV can be calculated as the difference between the FVC (or VC) and IC. While this value is inaccurate in absolute terms, it still allows one to examine the pattern of change in operating volumes [9, 50, 51]. How an investigator chooses to express their operating volumes (litres, %TLC, %TLC_pred_, etc.) depends on their preference, the nature of their clinical/research question, and whether or not there are group comparisons involved. For example, if a comparison is made between healthy individuals and patients with lung disease, then expressing the data as a percentage of predicted TLC may give more insight into the effects of disease (e.g., static lung hyperinflation) than if the data are expressed as a percentage of the measured TLC, which could be abnormal. Regardless of the approach, the pattern of change in EELV and EILV will be the same. Combining operating lung volume data with breathing pattern responses (e.g., *V*_*T*_ and breathing frequency) permits a more comprehensive evaluation of ventilatory limitation during exercise (Figure 4). 

## 5. Interpretation of IC Measurements

### 5.1. Typical Responses in Health and Disease

In the untrained healthy individual, systemic O_2_ transport, and not the ventilatory system, is the proximate limiting factor for maximal *V*_O_2__. The majority of studies in health have demonstrated that EELV decreases (IC increases) during most exercise intensities [50, 52–54] while a few have shown that it remains relatively constant [22, 55]. Those studies that demonstrated a decrease in EELV also showed considerable interindividual variability with some individuals decreasing EELV only at the highest exercise levels [54]. However, in most individuals, the changes are progressive with increasing exercise intensity. Typically, *V*_*T*_ expands to reach its maximal value at ~70% of the IC (i.e., when dynamic IRV is 0.5–1 L below TLC). In many untrained healthy individuals, this usually occurs near the limits of tolerance close to peak *V*_*E*_; a discreet inflection or plateau in the *V*_*T*_/*V*_*E*_ relation may not be discernible. In health, expiratory muscle recruitment during exercise results in reductions of EELV, which allow *V*_*T*_ to expand within the linear portion of the respiratory system's pressure-volume relation. Thus, earlier encroachment of EILV on the upper “stiffer” portion of this relation is avoided. In addition, vigorous expiratory muscle contraction stores energy in the chest wall, which is released during early inspiration, thereby assisting the inspiratory muscles [56, 57]. This strategy, together with breathing pattern adjustments, allows healthy individuals to increase *V*_*E*_ during exercise (up to 20 times resting values) without experiencing significant respiratory discomfort. 

This effective strategy to optimize respiratory muscle function and respiratory sensation during exercise in health is undermined in a number of clinical conditions characterized by airway dysfunction. In these situations, lung emptying is compromised by mechanical time constant (product of resistance and compliance) abnormalities in heterogeneously distributed alveolar units. Under these circumstances, the time available during spontaneous expiration is insufficient to allow EELV to decline to its natural relaxation volume, resulting in gas trapping or dynamic lung hyperinflation. Thus, a failure to decrease EELV, or an actual increase in EELV during exercise, has been shown in conditions where there is a combination of expiratory flow limitation and increased ventilatory requirements (e.g., natural aging, COPD, and cystic fibrosis). In healthy elderly individuals, changes in the lung connective tissue matrix result in increased lung compliance, which predisposes these individuals to expiratory flow limitation and gas trapping at higher ventilations during exercise [45, 58]. The ability to reduce EELV during exercise is also limited in individuals with a reduced resting expiratory reserve volume and EELV; in such patients, resting pulmonary function tests are otherwise normal (e.g., obesity [34], pregnancy [59], and in some patients with pulmonary arterial hypertension [37]). It should be noted that in these conditions, the resting IC is preserved, or actually increased, and the negative mechanical and sensory consequences of dynamic hyperinflation are likely to be less pronounced than when the resting IC is diminished.

The regulation of EELV in patients with chronic lung disease can be remarkably different from their healthy counterparts. In COPD, the resting IC, an indirect marker of lung hyperinflation, is an important predictor of peak *V*_*E*_ during symptom-limited exercise [16, 17, 60]. The lower the IC, the lower the *V*_*E*_ at which *V*_*T*_ reaches its plateau (or maximal value) having reached the minimal dynamic IRV [12]. At this point, there is a corresponding increase in breathing frequency. The effect of declining IC on breathing pattern and ventilatory capacity across the continuum of health and COPD is illustrated in Figure 4. Note that significant dynamic hyperinflation is detectable even in patients with milder COPD [61, 62]. The majority (80%–85%) of patients with moderate-to-severe COPD increase EELV (decrease IC) relative to resting values, even during submaximal exercise intensities [17, 33, 63, 64]. It is unclear why a minority of patients with COPD do not dynamically hyperinflate during exercise, but it may be related, at least in part, to having a lower resting IC [17, 64]. Smaller studies using optoelectronic plethysmography have identified varied behaviour of end-expiratory chest wall motion during exercise and have designated subgroups of COPD as nonhyperinflators (“euvolumics”) [7], and “early” and “late” hyperinflators [65]. The physiological consequences of dynamic hyperinflation are briefly summarized in Table 1 [21].

The sensory consequences will vary with the resting IC as this will determine the *V*_*E*_ at which the *V*_*T*_ reaches its maximal value. This *V*_*T*_ inflection, or plateau, which occurs at an IRV of 0.5–1.0 L below TLC (Figure 4), is an important mechanical event during exercise in COPD. This event marks the beginning of an ever widening disparity between central neural drive and the mechanical/muscular response of the respiratory system (i.e., neuromechanical uncoupling) [66]. At this point, dyspnea intensity escalates sharply towards intolerable levels and the distressing sensation of “unsatisfied inspiration” displaces “increased breathing effort” as the dominant qualitative descriptor [67]. Recent studies have suggested that dyspnea intensity during exercise in COPD is more closely associated with the increase in EILV (or the decrease in dynamic IRV) than with the increase in EELV, *per se* [64].

## 6. Effects of Selected Therapeutic Interventions on IC

### 6.1. Bronchodilators

Bronchodilators act to reduce airway smooth muscle tone, improve airway conductance, and accelerate the time constants for lung emptying of heterogeneously distributed alveolar units. Bronchodilators of all classes have consistently been shown to increase the resting IC in patients with COPD by an average of ~0.3 L (or 15%) (for review see [21]). This improvement reflects a decrease in resting lung hyperinflation and is associated with improvements in dyspnea and exercise endurance time [10, 14, 43, 68, 69]. Combining a long-acting anticholinergic with a long-acting *β*_2_ agonist may also have additive effects on improving IC [70]. The combination of an inhaled corticosteroid with a bronchodilator has also shown beneficial effects on resting IC compared with placebo [71]. 

**Figure 5:**
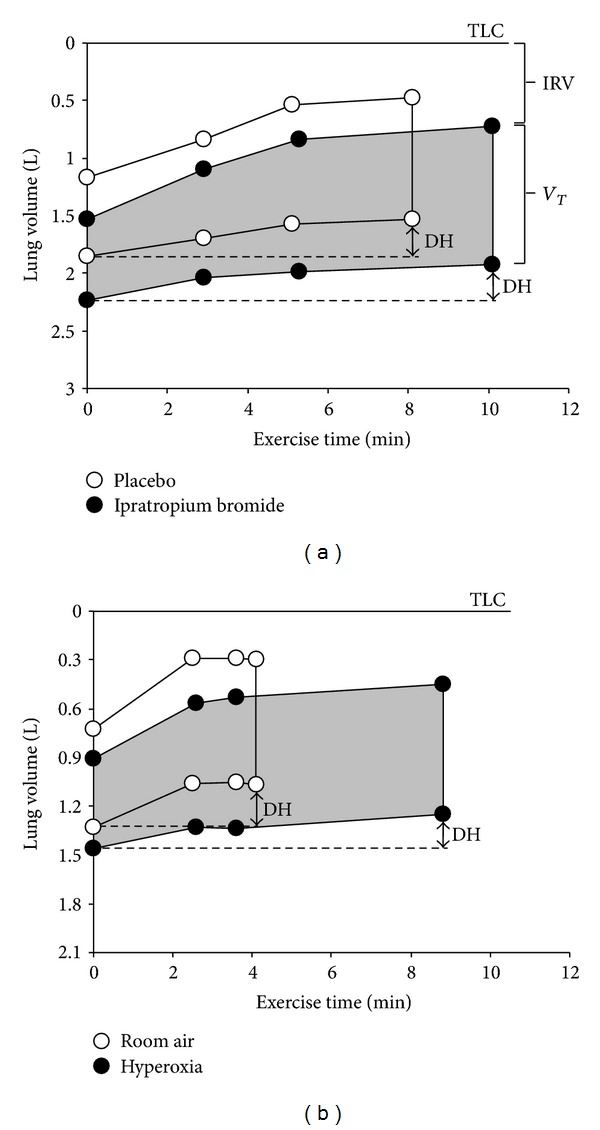
Operating lung volume plots during constant work rate cycle exercise in COPD patients following acute high-dose anticholinergic therapy versus placebo (a) and hyperoxia versus room air (b). The operating lung volumes (i.e., end-expiratory and end-inspiratory lung volumes) were reduced at rest (0 min) following anticholinergic therapy and resulted in a downward shift in operating lung volumes throughout exercise. The magnitude of dynamic hyperinflation at peak exercise (calculated as the difference in EELV from resting values) did not change following bronchodilation. The magnitude of dynamic hyperinflation was also similar at peak exercise (~0.2 L) during hyperoxia compared with room air in hypoxemic patients with COPD. Data for these graphs are based on previously published studies from our laboratory [43, 74]. IRV, inspiratory reserve volume; *V*_*T*_, tidal volume; DH, dynamic hyperinflation; TLC, total lung capacity.

The effects of bronchodilators and various forms of combination therapy also increase IC during exercise [10, 14, 43, 68, 69]. This increase in IC reflects a reciprocal decrease in EELV (Figure 5(a)) and, thus, it is commonly thought that pharmacotherapy reduces dynamic hyperinflation. However, bronchodilators, alone or in combination with inhaled corticosteroids, rarely reduce the absolute magnitude of dynamic hyperinflation that occurs acutely during exercise. In fact, the magnitude of dynamic hyperinflation either remains the same or may worsen slightly reflecting the higher ventilations that can be achieved during exercise as a result of the bronchodilation [43, 69, 72]. The reason for this misconception is based on the fact that we do not currently have an established operational definition of dynamic hyperinflation. Traditionally, dynamic hyperinflation is defined as an increase in EELV (or decrease in IC) relative to *resting* values. The reason pharmacotherapy does not reduce dynamic hyperinflation, based on this definition, is because the resting EELV (and IC) also improves with bronchodilation. In other words, bronchodilator treatment or combination therapy simply cause a parallel downward shift in the EELV over the course of the exercise test reflecting the reduction in resting (static) lung hyperinflation (Figure 5(a)). Regardless of the terminology, we can confidently say that improving airway function with pharmacotherapy has beneficial effects on IC at rest, and therefore during exercise. This increase in IC delays the onset of critical ventilatory constraints to ventilation. Improvements in dyspnea and exercise tolerance are closely related with release of *V*_*T*_ restriction and enhanced neuromechanical coupling of the respiratory system [66]. 

### 6.2. Oxygen

A number of studies have shown improvements in exercise performance and reductions in exertional dyspnea in response to hyperoxic breathing in patients with COPD [31, 73–75]. The underlying mechanisms of dyspnea relief and enhanced exercise performance with hyperoxia are controversial [73, 76–78] but are likely related, in part, to lower ventilatory requirements [31, 74, 77] due to reduced chemoreceptor drive [73, 75]. Given that dynamic hyperinflation is largely determined by *V*_*E*_, it seems intuitive that hyperoxic breathing would improve the IC during exercise and, thus, reduce the magnitude (or delay the onset) of dynamic hyperinflation. However, the interrelationship between possible reductions in dynamic hyperinflation and improvements in dyspnea and exercise endurance with hyperoxia has been difficult to establish. O'Donnell et al. [74] evaluated the effects of hyperoxic breathing during exercise in hypoxemic COPD patients and demonstrated a significant delay in dynamic hyperinflation during exercise compared with room air. However, the magnitude of dynamic hyperinflation at peak exercise was unaffected by hyperoxia (Figure 5(b)), which is consistent with the recent work of Eves et al. [79]. It should be noted that the beneficial effects of delaying dynamic hyperinflation and reducing operating lung volumes during hyperoxic exercise may be less pronounced in normoxic or mildly hypoxemic COPD patients [72, 77]. 

A study by Somfay et al. [31] evaluated the dose-response effects of hyperoxia on operating lung volumes during exercise in normoxic COPD patients and in healthy controls. Their study demonstrated consistent increases in IC as the fraction of inspired O_2_ increased from 0.21 to 0.50 with no further improvements thereafter in the COPD patients (no effect was observed in the healthy controls). The improvement in dyspnea with hyperoxia was correlated with changes in both EELV and EILV. However, this relationship has not been found in more recent studies [72, 80]. Collectively, these studies suggest that hyperoxia consistently reduces *V*_*E*_ and dyspnea and improves exercise tolerance in patients with COPD. Most studies show some favourable effect of hyperoxia on IC during submaximal exercise but responses are highly variable and are likely dependent on the baseline level of respiratory impairment (e.g., resting level of hyperinflation, airway obstruction, and hypoxemia; hyperinflator versus nonhyperinflator during exercise, etc.) [72, 74, 77, 80]. 

### 6.3. Exercise Training

Well-designed exercise training interventions as part of a pulmonary rehabilitation program can improve exercise performance to a greater extent than other available treatment interventions for patients with COPD [81]. One of the primary mechanisms by which exercise training can improve exercise capacity is through a reduction in ventilatory stimulation due to lower levels of lactic acidosis (and *V*_CO_2__) for any given exercise intensity [82]. The reduction in ventilation following exercise training seems to be mediated primarily through a reduced breathing frequency [83, 84]. This permits greater time for expiration between breaths, and, like other interventions that reduce ventilation (e.g., oxygen), this should have some favourable effects on IC during exercise. However, the impact of exercise training on IC behaviour during cycle exercise has been both modest and inconsistent across studies and it is clear that improvement in IC during exercise is not obligatory to achieve important improvements in the intensity and affective domains of dyspnea following exercise training [83–88].

## 7. Conclusion

Calculation of the peak exercise *V*_*E*_ to MVV ratio has traditionally been used to evaluate ventilatory reserve during CPET. This provides an estimate of demand versus capacity but gives little information on the source or nature of the ventilatory impairment. The resting IC provides valuable information on potential ventilatory capacity during exercise. A low IC increases the likelihood of critical dynamic mechanical constraints at relatively low exercise intensities, thus limiting further increases in ventilation. Examination of the IC, IRV, and breathing pattern at a standardized time or ventilation during exercise gives important insight into the individual's prevailing mechanical abnormalities and the mechanisms underlying dyspnea and exercise limitation. This detailed approach to CPET interpretation can also give valuable insight into the mechanisms of dyspnea relief and exercise performance improvements following various therapeutic interventions. The wealth of data derived from IC measurements also allows detection of physiological impairment in dyspneic patients with near-normal spirometry (e.g., mild COPD, pulmonary arterial hypertension, obesity, etc.) and may prompt specific treatment interventions to improve exercise tolerance. Collectively, the valuable information gained from the IC and derived physiological parameters provide a solid rationale for their regular inclusion during standard CPET for both clinical and research purposes.

## Figures and Tables

**Table 1:** Consequences of dynamic hyperinflation.

Negative consequences of dynamic hyperinflation	
(i) Increased elastic and threshold loading on the inspiratory muscles	
(ii) Increased work and O_2_ cost of breathing	
(iii) Functional inspiratory muscle weakness and possible fatigue	
(iv) Mechanical constraint on tidal volume expansion	
(v) Early ventilatory limitation to exercise	
(vi) Increased neuromechanical uncoupling of the respiratory system	
(vii) CO_2_ retention and possibly arterial hypoxemia	
(viii) Potential adverse cardiovascular consequences	
(ix) Increased dyspnea and exercise intolerance	

For a more detailed review on the consequences of dynamic hyperinflation, see O'Donnell and Laveneziana [21].

**Table 2:** Data derived from IC maneuvers.

Variable	Formula	Units
ΔIC	IC − IC_rest_	L
IC/TLC	(IC/TLC) × 100	%
IC/VC	(IC/VC) × 100	%
IRV	IC − *V*_*T*_ or TLC − EILV	L, %TLC, %TLC_pred _
*V*_*T*_/IC	(*V*_*T*_/IC) × 100	%
EELV	TLC − IC	L, %TLC, %TLC_pred_,
	*Surrogates for EELV:*	
	FVC − IC VC − IC	%FVC, %FVC_pred_, %VC, %VC_pred_
EILV	EELV + *V*_*T*_	L, %TLC, %TLC_pred_, %FVC, %FVC_pred_, %VC, %VC_pred_
EFL	(*V*_FL_/*V*_*T*_) × 100	%

IC: inspiratory capacity; ΔIC: change in inspiratory capacity from rest; TLC: total lung capacity; VC: vital capacity; IRV: inspiratory reserve volume; *V*_*T*_: tidal volume; EILV: end-inspiratory lung volume; TLC_pred_: predicted total lung capacity; EELV: end-expiratory lung volume; FVC: forced vital capacity; FVC_pred_: predicted forced vital capacity; VC_pred_: predicted vital capacity; EFL: expiratory flow limitation; *V*_FL_: volume of tidal breath that is flow limited on expiration (see Figure 1(a)).

## References

[1] American Thoracic Society and American College of Chest Physicians (2003). ATS/ACCP Statement on cardiopulmonary exercise testing. *American Journal of Respiratory and Critical Care Medicine*.

[2] Klas JV, Dempsey JA (1989). Voluntary versus reflex regulation of maximal exercise flow: volume loops. *American Review of Respiratory Disease*.

[3] Johnson BD, Weisman IM, Zeballos RJ, Beck KC (1999). Emerging concepts in the evaluation of ventilatory limitation during exercise: the exercise tidal flow-volume loop. *Chest*.

[4] Guenette JA, Dominelli PB, Reeve SS, Durkin CM, Eves ND, Sheel AW (2010). Effect of thoracic gas compression and bronchodilation on the assessment of expiratory flow limitation during exercise in healthy humans. *Respiratory Physiology & Neurobiology*.

[5] Johnson BD, Seow KC, Pegelow DF, Dempsey JA (1990). Adaptation of the inert gas FRC technique for use in heavy exercise. *Journal of Applied Physiology*.

[6] Clarenbach CF, Senn O, Brack T, Kohler M, Bloch KE (2005). Monitoring of ventilation during exercise by a portable respiratory inductive plethysmograph. *Chest*.

[7] Aliverti A, Stevenson N, Dellacà RL, Lo Mauro A, Pedotti A, Calverley PMA (2004). Regional chest wall volumes during exercise in chronic obstructive pulmonary disease. *Thorax*.

[8] Johnson BD, Beck KC, Olson LJ (2000). Ventilatory constraints during exercise in patients with chronic heart failure. *Chest*.

[9] Guenette JA, Witt JD, McKenzie DC, Road JD, Sheel AW (2007). Respiratory mechanics during exercise in endurance-trained men and women. *Journal of Physiology*.

[10] O’Donnell DE, Lam M, Webb KA (1998). Measurement of symptoms, lung hyperinflation, and endurance during exercise in chronic obstructive pulmonary disease. *American Journal of Respiratory and Critical Care Medicine*.

[11] McClaran SR, Harms CA, Pegelow DF, Dempsey JA (1998). Smaller lungs in women affect exercise hyperpnea. *Journal of Applied Physiology*.

[12] O'Donnell DE, Guenette JA, Maltais F, Webb KA (2012). Decline of resting inspiratory capacity in COPD: the impact on breathing pattern, dyspnea, and ventilatory capacity during exercise. *Chest*.

[13] Di Marco F, Milic-Emili J, Boveri B (2003). Effect of inhaled bronchodilators on inspiratory capacity and dyspnoea at rest in COPD. *European Respiratory Journal*.

[14] O’Donnell DE, Flüge T, Gerken F (2004). Effects of tiotropium on lung hyperinflation, dyspnoea and exercise tolerance in COPD. *European Respiratory Journal*.

[15] Celli B, ZuWallack R, Wang S, Kesten S (2003). Improvement in resting inspiratory capacity and hyperinflation with tiotropium in COPD patients with increased static lung volumes. *Chest*.

[16] Albuquerque ALP, Nery LE, Villaça DS (2006). Inspiratory fraction and exercise impairment in COPD patients GOLD stages II-III. *European Respiratory Journal*.

[17] O’Donnell DE, Revill SM, Webb KA (2001). Dynamic hyperinflation and exercise intolerance in chronic obstructive pulmonary disease. *American Journal of Respiratory and Critical Care Medicine*.

[18] O’Donnell DE, D’Arsigny C, Fitzpatrick M, Webb KA (2002). Exercise hypercapnia in advanced chronic obstructive pulmonary disease: the role of lung hyperinflation. *American Journal of Respiratory and Critical Care Medicine*.

[19] Casanova C, Cote C, De Torres JP (2005). Inspiratory-to-total lung capacity ratio predicts mortality in patients with chronic obstructive pulmonary disease. *American Journal of Respiratory and Critical Care Medicine*.

[20] Zaman M, Mahmood S, Altayeh A (2010). Low inspiratory capacity to total lung capacity ratio is a risk factor for chronic obstructive pulmonary disease exacerbation. *American Journal of the Medical Sciences*.

[21] O’Donnell DE, Laveneziana P (2006). The clinical importance of dynamic lung hyperinflation in COPD. *COPD: Journal of Chronic Obstructive Pulmonary Disease*.

[22] Stubbing DG, Pengelly LD, Morse JLC, Jones NL (1980). Pulmonary mechanics during exercise in normal males. *Journal of Applied Physiology*.

[23] Stubbing DG, Pengelly LD, Morse JLC, Jones NL (1980). Pulmonary mechanics during exercise in subjects with chronic airflow obstruction. *Journal of Applied Physiology*.

[24] Sinderby C, Spahija J, Beck J (2001). Diaphragm activation during exercise in chronic obstructive pulmonary disease. *American Journal of Respiratory and Critical Care Medicine*.

[25] Bellemare F, Grassino A (1983). Force reserve of the diaphragm in patients with chronic obstructive pulmonary disease. *Journal of Applied Physiology*.

[26] Yan S, Kaminski D, Sliwinski P (1997). Reliability of inspiratory capacity for estimating end-expiratory lung volume changes during exercise in patients with chronic obstructive pulmonary disease. *American Journal of Respiratory and Critical Care Medicine*.

[27] Dolmage TE, Goldstein RS (2002). Repeatability of inspiratory capacity during incremental exercise in patients with severe COPD. *Chest*.

[28] Belman MJ, Botnick WC, Shin JW (1996). Inhaled bronchodilators reduce dynamic hyperinflation during exercise in patients with chronic obstructive pulmonary disease. *American Journal of Respiratory and Critical Care Medicine*.

[29] Martinez FJ, De Oca MM, Whyte RI, Stetz J, Gay SE, Celli BR (1997). Lung-volume reduction improves dyspnea, dynamic hyperinflation, and respiratory muscle function. *American Journal of Respiratory and Critical Care Medicine*.

[30] O’Donnell DE, Webb KA, Bertley JC, Chau LKL, Conlan AA (1996). Mechanisms of relief of exertional breathlessness following unilateral bullectomy and lung volume reduction surgery in emphysema. *Chest*.

[31] Somfay A, Porszasz J, Lee SM, Casaburi R (2001). Dose-response effect of oxygen on hyperinflation and exercise endurance in nonhypoxaemic COPD patients. *European Respiratory Journal*.

[32] Palange P, Valli G, Onorati P (2004). Effect of heliox on lung dynamic hyperinflation, dyspnea, and exercise endurance capacity in COPD patients. *Journal of Applied Physiology*.

[33] O’Donnell DE, Travers J, Webb KA (2009). Reliability of ventilatory parameters during cycle ergometry in multicentre trials in COPD. *European Respiratory Journal*.

[34] Ofir D, Laveneziana P, Webb KA, O’Donnell DE (2007). Ventilatory and perceptual responses to cycle exercise in obese women. *Journal of Applied Physiology*.

[35] O’Donnell DE, D’Arsigny C, Raj S, Abdollah H, Webb KA (1999). Ventilatory assistance improves exercise endurance in stable congestive heart failure. *American Journal of Respiratory and Critical Care Medicine*.

[36] Laveneziana P, O’Donnell DE, Ofir D (2009). Effect of biventricular pacing on ventilatory and perceptual responses to exercise in patients with stable chronic heart failure. *Journal of Applied Physiology*.

[37] Richter MJ, Voswinckel R, Tiede H (2012). Dynamic hyperinflation during exercise in patients with precapillary pulmonary hypertension. *Respiratory Medicine*.

[38] Alison JA, Regnis JA, Donnelly PM, Adams RD, Sullivan CE, Bye PTP (1998). End-expiratory lung volume during arm and leg exercise in normal subjects and patients with cystic fibrosis. *American Journal of Respiratory and Critical Care Medicine*.

[39] Yeh MP, Adams TD, Gardner RM, Yanowitz FG (1984). Effect of O_2_, N_2_, and CO_2_ composition on nonlinearity of Fleisch pneumotachograph characteristics. *Journal of Applied Physiology*.

[40] Miller MR, Hankinson J, Brusasco V (2005). Standardisation of spirometry. *European Respiratory Journal*.

[41] Pellegrino R, Rodarte JR, Brusasco V (1998). Assessing the reversibility of airway obstruction. *Chest*.

[42] American Association for Respiratory Care AARC guideline: body plethysmography: 2001 revision & update. *Respiratory Care*.

[43] O’Donnell DE, Lam M, Webb KA (1999). Spirometric correlates of improvement in exercise performance after anticholinergic therapy in chronic obstructive pulmonary disease. *American Journal of Respiratory and Critical Care Medicine*.

[44] Berton DC, Reis M, Siqueira ACB (2010). Effects of tiotropium and formoterol on dynamic hyperinflation and exercise endurance in COPD. *Respiratory Medicine*.

[45] Ofir D, Laveneziana P, Webb KA, Lam YM, O’Donnell DE (2008). Sex differences in the perceived intensity of breathlessness during exercise with advancing age. *Journal of Applied Physiology*.

[46] Hsia D, Casaburi R, Pradhan A, Torres E, Porszasz J (2009). Physiological responses to linear treadmill and cycle ergometer exercise in COPD. *European Respiratory Journal*.

[47] Holm SM, Rodgers WM, Haennel RG (2011). Physiological responses to treadmill and cycle ergometer exercise testing in chronic obstructive pulmonary disease. *American Journal of Respiratory and Critical Care Medicine*.

[48] Babb TG, Viggiano R, Hurley B, Staats B, Rodarte JR (1991). Effect of mild-to-moderate airflow limitation on exercise capacity. *Journal of Applied Physiology*.

[49] Bauerle O, Chrusch CA, Younes M (1998). Mechanisms by which COPD affects exercise tolerance. *American Journal of Respiratory and Critical Care Medicine*.

[50] Mota S, Casan P, Drobnic F (1999). Expiratory flow limitation during exercise in competition cyclists. *Journal of Applied Physiology*.

[51] Wilkie SS, Guenette JA, Dominelli PB, Sheel AW (2012). Effects of an aging pulmonary system on expiratory flow limitation and dyspnoea during exercise in healthy women. *European Journal of Applied Physiology*.

[52] Henke KG, Sharratt M, Pegelow D, Dempsey JA (1988). Regulation of end-expiratory lung volume during exercise. *Journal of Applied Physiology*.

[53] Johnson BD, Saupe KW, Dempsey JA (1992). Mechanical constraints on exercise hyperpnea in endurance athletes. *Journal of Applied Physiology*.

[54] Sharratt MT, Henke KG, Aaron EA, Pegelow DF, Dempsey JA (1987). Exercise-induced changes in functional residual capacity. *Respiration Physiology*.

[55] Kiers A, van der Mark TW, Woldring MG, Peset R (1980). Determination of the functional residual capacity during exercise. *Ergonomics*.

[56] Collett PW, Engel LA (1986). Influence of lung volume on oxygen cost of resistive breathing. *Journal of Applied Physiology*.

[57] Road J, Newman S, Derenne JP, Grassino A (1986). In vivo length-force relationship of canine diaphragm. *Journal of Applied Physiology*.

[58] Johnson BD, Reddan WG, Seow KC, Dempsey JA (1991). Mechanical constraints on exercise hyperpnea in a fit aging population. *American Review of Respiratory Disease*.

[59] Jensen D, Webb KA, Davies GAL, O’Donnell DE (2008). Mechanical ventilatory constraints during incremental cycle exercise in human pregnancy: implications for respiratory sensation. *Journal of Physiology*.

[60] Diaz O, Villafranca C, Ghezzo H (2000). Role of inspiratory capacity on exercise tolerance in COPD patients with and without tidal expiratory flow limitation at rest. *European Respiratory Journal*.

[61] Ofir D, Laveneziana P, Webb KA, Lam YM, O’Donnell DE (2008). Mechanisms of dyspnea during cycle exercise in symptomatic patients with GOLD stage I chronic obstructive pulmonary disease. *American Journal of Respiratory and Critical Care Medicine*.

[62] Guenette JA, Jensen D, Webb KA, Ofir D, Raghavan N, O’Donnell DE (2011). Sex differences in exertional dyspnea in patients with mild COPD: physiological mechanisms. *Respiratory Physiology & Neurobiology*.

[63] Garcia-Rio F, Lores V, Mediano O (2009). Daily physical activity in patients with chronic obstructive pulmonary disease is mainly associated with dynamic hyperinflation. *American Journal of Respiratory and Critical Care Medicine*.

[64] Guenette JA, Webb KA, O'Donnell DE (2012). Does dynamic hyperinflation contribute to dyspnoea during exercise in patients with COPD?. *European Respiratory Journal*.

[65] Vogiatzis I, Georgiadou O, Golemati S (2005). Patterns of dynamic hyperinflation during exercise and recovery in patients with severe chronic obstructive pulmonary disease. *Thorax*.

[66] O’Donnell DE, Hamilton AL, Webb KA (2006). Sensory-mechanical relationships during high-intensity, constant-work-rate exercise in COPD. *Journal of Applied Physiology*.

[67] Laveneziana P, Webb KA, Ora J, Wadell K, O'Donnell DE (2011). Evolution of dyspnea during exercise in chronic obstructive pulmonary disease: impact of critical volume constraints. *American Journal of Respiratory and Critical Care Medicine*.

[68] Maltais F, Hamilton A, Marciniuk D (2005). Improvements in symptom-limited exercise performance over 8 h with once-daily tiotropium in patients with COPD. *Chest*.

[69] O’Donnell DE, Voduc N, Fitzpatrick M, Webb KA (2004). Effect of salmeterol on the ventilatory response to exercise in chronic obstructive pulmonary disease. *European Respiratory Journal*.

[70] van Noord JA, Aumann JL, Janssens E (2006). Effects of tiotropium with and without formoterol on airflow obstruction and resting hyperinflation in patients with COPD. *Chest*.

[71] O’Donnell DE, Sciurba F, Celli B (2006). Effect of fluticasone propionate/salmeterol on lung hyperinflation and exercise endurance in COPD. *Chest*.

[72] Peters MM, Webb KA, O’Donnell DE (2006). Combined physiological effects of bronchodilators and hyperoxia on exertional dyspnoea in normoxic COPD. *Thorax*.

[73] Dean NC, Brown JK, Himelman RB, Doherty JJ, Gold WM, Stulbarg MS (1992). Oxygen may improve dyspnea and endurance in patients with chronic obstructive pulmonary disease and only mild hypoxemia. *American Review of Respiratory Disease*.

[74] O’Donnell DE, D’Arsigny C, Webb KA (2001). Effects of hyperoxia on ventilatory limitation during exercise in advanced chronic obstructive pulmonary disease. *American Journal of Respiratory and Critical Care Medicine*.

[75] Stein DA, Bradley BL, Miller WC (1982). Mechanisms of oxygen effects on exercise in patients with chronic obstructive pulmonary disease. *Chest*.

[76] Lane R, Cockcroft A, Adams L, Guz A (1987). Arterial oxygen saturation and breathlessness in patients with chronic obstructive airways disease. *Clinical Science*.

[77] O’Donnell DE, Bain DJ, Webb KA (1997). Factors contributing to relief of exertional breathlessness during hyperoxia in chronic airflow limitation. *American Journal of Respiratory and Critical Care Medicine*.

[78] Swinburn CR, Wakefield JM, Jones PW (1984). Relationship between ventilation and breathlessness during exercise in chronic obstructive airways disease is not altered by prevention of hypoxaemia. *Clinical Science*.

[79] Eves ND, Petersen SR, Haykowsky MJ, Wong EY, Jones RL (2006). Helium-hyperoxia, exercise, and respiratory mechanics in chronic obstructive pulmonary disease. *American Journal of Respiratory and Critical Care Medicine*.

[80] Bruni GI, Gigliotti F, Binazzi B, Romagnoli I, Duranti R, Scano G (2012). Dyspnea, chest wall hyperinflation, and rib cage distortion in exercising patients with chronic obstructive pulmonary disease. *Medicine and Science in Sports and Exercise*.

[81] Troosters T, Casaburi R, Gosselink R, Decramer M (2005). Pulmonary rehabilitation in chronic obstructive pulmonary disease. *American Journal of Respiratory and Critical Care Medicine*.

[82] Casaburi R, Patessio A, Ioli F, Zanaboni S, Donner CF, Wasserman K (1991). Reductions in exercise lactic acidosis and ventilation as a result of exercise training in patients with obstructive lung disease. *American Review of Respiratory Disease*.

[83] Porszasz J, Emtner M, Goto S, Somfay A, Whipp BJ, Casaburi R (2005). Exercise training decreases ventilatory requirements and exercise-induced hyperinflation at submaximal intensities in patients with COPD. *Chest*.

[84] O’Donnell DE, McGuire M, Samis L, Webb KA (1998). General exercise training improves ventilatory and peripheral muscle strength and endurance in chronic airflow limitation. *American Journal of Respiratory and Critical Care Medicine*.

[85] Pellegrino R, Villosio C, Milanese U, Garelli G, Rodarte JR, Brusasco V (1999). Breathing during exercise in subjects with mild-to-moderate airflow obstruction: effects of physical training. *Journal of Applied Physiology*.

[86] Gigliotti F, Coli C, Bianchi R (2003). Exercise training improves exertional dyspnea in patients with COPD: evidence of the role of mechanical factors. *Chest*.

[87] Puente-Maestu L, Abad YM, Pedraza F, Sánchez G, Stringer WW (2006). A controlled trial of the effects of leg training on breathing pattern and dynamic hyperinflation in severe COPD. *Lung*.

[88] Wadell K, Webb KA, Preston ME Impact of pulmonary rehabilitation on the major dimensions of dyspnea in COPD.

